# An Epidemiological Perspective on the Investigation of Genocide

**DOI:** 10.3389/fepid.2022.844895

**Published:** 2022-03-03

**Authors:** Peter Tammes

**Affiliations:** Bristol Medical School (Population Health Sciences), University of Bristol, Bristol, United Kingdom

**Keywords:** genocide, violence, mortality, Holocaust, Rwanda, epidemiology

## Introduction

The World Health Assembly adopted in 1996 a resolution declaring violence a leading worldwide public health problem which was followed by the launch of a World Health Organization (WHO) campaign on violence prevention in 2002 (1). The WHO divides violence into self-directed violence, interpersonal violence, and collective violence. Related to the latter, population, and epidemiological studies investigated patterns and causes of deaths, including mass slaughtering, during and after armed conflicts such as in Darfur (2, 3) and Kosovo (4), but hardly for genocide-related killings such as the killing of Tutsi in Rwanda, which took place between April 7 and mid-July 1994, and the killing of Jews during World War II (WWII) or the Holocaust. Genocide is the intent of destroying in whole or in part the way of life and existence of a population group whether through acts of total war, racial extinction, or ethnic cleansing (5). This Opinion's aim is to raise attention to genocide-related deaths, an under-researched subject within the field of epidemiology and population and public health studies. It will do this by applying epidemiological measures to contribute to a better understanding of the killings during the Rwandan genocide and the Holocaust as case studies.

Often, the Rwanda genocide is compared with the Holocaust. For example, in Adam Jones' *Genocide* (6), a detailed and comprehensive textbook in the field of genocide studies, it is mentioned that on 20 April 1994 “between thirty-five and forty-three thousand people died in less than six hours. This was more than were killed in the Nazis' two-day slaughters of Jews outside Odessa and Kiev (at Babi Yar) in 1941, or in the largest single-day extermination spree in the gas chambers of Auschwitz-Birkenau” (p. 482). Furthermore, it refers to Prunier's statement that “the daily killing rate was at least five times that of the Nazi death camps” (p. 473). It is unclear how Prunier (7) calculated this daily killing rate ratio. Nevertheless, many refer to this ratio.

In a recent article, Stone (8) provided a sample of typical comparisons between the Holocaust and the Rwandan genocide by scholars, human-rights advocates, and policymakers stating the Rwandan genocide is the “most intense” genocide of the twentieth century, or the “most rapid genocide ever recorded.” Contrary to these scholars, Stone stated that the killing of the Jews during the Holocaust was more intense than the Rwandan genocide. This statement is based on the calculation of a so-called “kill rate:” the number of victims murdered per time unit (8). Stone's study on the Nazi genocide focused on the killings during Operation Reinhard (March 1942–November 1943) in Nazi-camps Belzec, Sobibor, and Treblinka—about 1.7 million Jews from Nazi German-occupied Poland or the General Government, the killing in Nazi camp Auschwitz of about 800,000 Jews from other Nazi occupied areas (March 1942–November 1944), and the widespread shooting of about 360,000 Jews in Ukraine, South Russia, and Bialystok (August–November 1942). Stone identified hyper-extreme killings of Jews during August, September, and October 1942 and determined the “kill rate” for the Holocaust at 1.47 million over 100 days, or 14,700 per day. For the Rwandan genocide, Stone determined the “kill rate” at 800,000 over 100 days or 8,000 per day (8). These calculations suggest that the Holocaust “kill rate” is nearly twice as high as the Rwandan genocide “kill rate.” Though, on some specific days, such as on some days in April 1994 (6) or on some days in October 1942 (8), daily killings were much higher; the “kill rate” calculation depends then on the selected time-period. Besides, bigger targeted groups can have a higher “kill rate” simply because of their population size.

To better understand and help to solve this controversy, we include some other measures often used in epidemiology such as mortality and survival rates. We used rough estimates as given by Prunier on the Rwanda genocide; a recent article discussed the number of Tutsi deaths in more detail but that is beyond this Opinion article (9). Similarly, we used data presented in Stone's study on the Nazi genocide (8). Since Stone's study included only victims, data available on victims and survivors of the Nazi persecution of Jews in the Netherlands (10) are included to compare their survival rates with survival rates of Tutsis in Rwanda.

## Measuring Death Fequencies for the Rwandan Genocide and the Holocaust

### Mortality Rates

Within epidemiology, measures such as the “kill rate” are referred to as an absolute rate (11). When measuring death frequency, the mortality rate is helpful when comparing groups or populations. To measure mortality rate, one must take into account the time (i.e., months) persons lived till they were killed or had survived at the end of a certain time-period (i.e., end of war or genocide), contributing to the total person-months of observations. When applying this measure to the Nazi genocide and the Rwandan genocide, the Jewish and Tutsi populations are considered closed populations—hardly any members left, or new members entered. Data presented by Stone (8) for the Nazi genocide between March 1942 and November 1944 were used to calculate an estimated mortality rate of 85 Jews per 1,000 person-months. If we only focus on August-October 1942 a 3-month period with the highest numbers of killings—though still many survivors at the end of October 1942, the mortality rate is estimated at 223 Jews per 1,000 person-months. Rough estimates of Tutsi killings in spring 1994 provided by Prunier (7) were used to calculate an estimated mortality rate of 396 Tutsi per 1,000 person-months. The Rwandan genocide mortality rate is then about 4.5 times the mortality rate of the Nazi genocide and about 1.8 times the mortality rate for the 3-month period of hyper-extreme killings in 1942.

### Survival Curves

**Figure 1:**
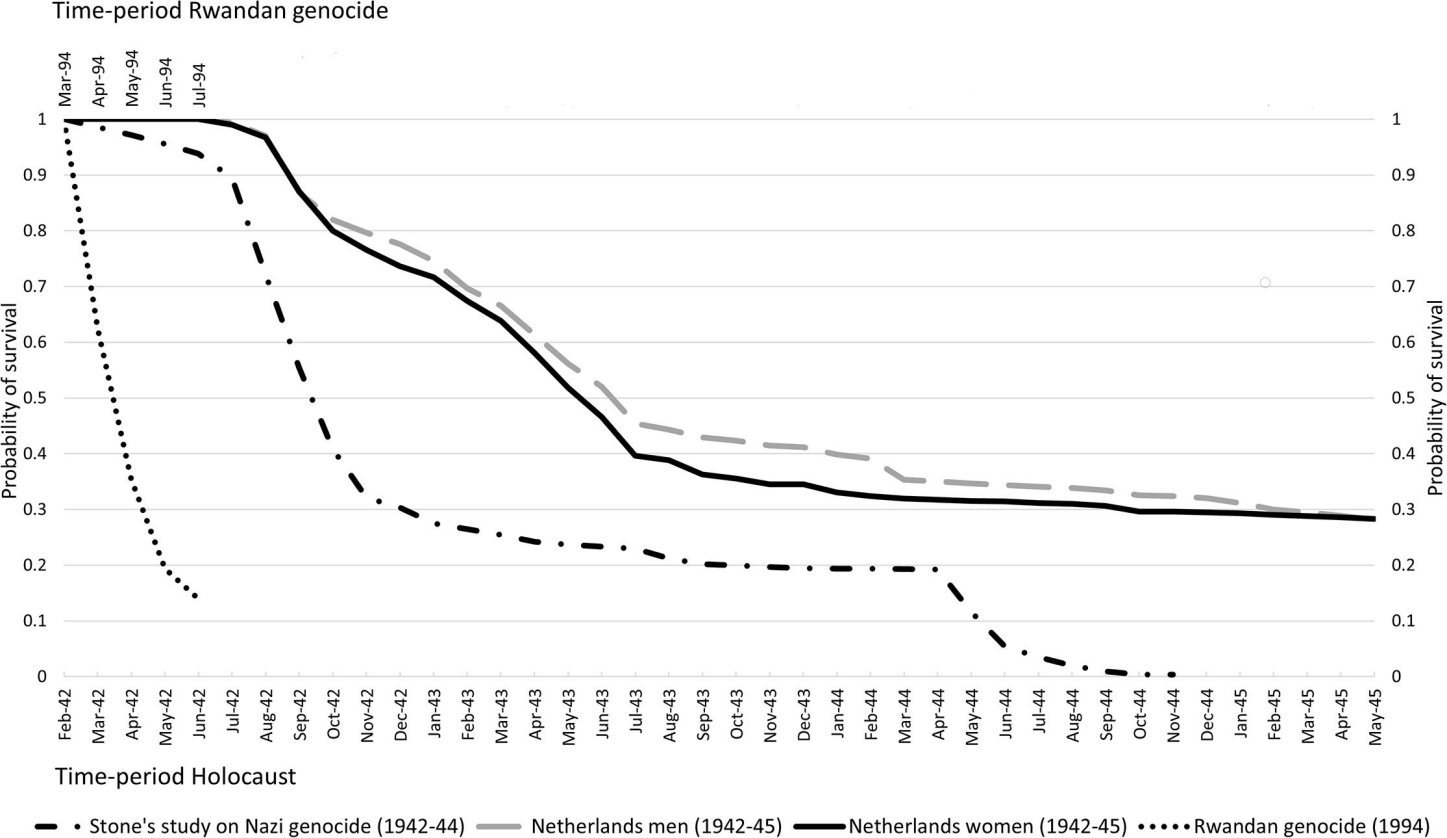
Survival curves for Tutsis during the Rwandan genocide, for Jews from the Netherlands during the Holocaust, and for Jews killed in Nazi-camps Belzec, Sobibor, Treblinka, Auschwitz and during shootings.

Since killings fluctuate over time, particularly in longer time periods of observation, it might be informative to calculate the cumulative proportion surviving or the survival curve. Figure 1 shows these curves based on data for the Rwandan genocide and Stone's study on the Nazi genocide; 95% confidence intervals (95%CI) are not included in this figure since these were very small.

As the genocide faced by the population of about 930,000 Tutsis living in Rwanda lasted about 3 months with a high number of deaths from the beginning onwards, the survival curve shows a steep decrease in probability of survival until the last month resulting in a cumulative proportion surviving Tutsis in Rwanda of 0.140 (95%CI 0.139–0.141). The killing of over 2.8 million Jews in camps Belzec, Sobibor, Treblinka, and Auschwitz and during shootings started in March 1942 and lasted 32 months. In summer and autumn of 1942, the number of deaths increased heavily resulting in a drop of the survival probability to 0.321 (95%CI 0.320–0.321) in November 1942; a drop of about 0.68 in 9 months' time. The Jewish survival curve for July–November 1942 shows nearly the same steep decrease as the Tutsis' survival curve for April–June 1994. The Jewish survival curve shows another sharp decline in May and June 1944, mainly due to increased killings in Auschwitz. There are hardly any Jewish survivors in November 1944 as nearly all deported to these four camps were killed.

Figure 1 also shows survival curves for about 117,000 Jews in the Netherlands−83% of all Jews, split by sex (10). These curves are based on available individual-level instead of aggregated data. The data contain information on date, place and causes of death. The systematic deportation of Jews from the Netherlands started in July 1942 and to reduce immortal-time bias (12) we have excluded Jews being caught and deported before that time in a few roundups aimed to catch Jewish young adult men. Though many were killed in camps after being deported, some died in Dutch transit-camp Westerbork, in hiding or from suicide while some others are assumed to have died of natural causes in the Netherlands. This latter cause of death can be seen as a “competing risk” and these cases have been censored when calculating survival rates. The survival curves for both sexes show a similar pattern, though the survival curve for women had dropped stronger by September 1943 (survival probability: 0.363; 95%CI 0.359–0.367) than that for men (0.429; 95%CI 0.425–0.433). Although the drop in survival for Jewish men and women identifies a period of intense deportations and killings, this drop is less steep than for Tutsis during the Rwandan genocide and the timespan of these intense killings was five times that in Rwanda (April–mid-July 1994). After September 1943, the survival curves show a smooth decrease in probability of survival, resulting in similar cumulative proportions surviving WWII for Jewish men (survival probability: 0.281; 95%CI 0.278–0.285) and women (survival probability: 0.283; 95%CI 0.279–0.287) from the Netherlands. Some of these Jews survived Nazi camps such as Auschwitz, Bergen-Belsen or Theresienstadt while others survived by being exempted from deportation and/or by hiding, fleeing or reclassification of their Jewish status.

## Profiles of Genocide-Related Deaths

The survival curves of these cases indicate different epidemiological profiles of genocide-related deaths. The mortality rate and survival curve for Rwanda indicate an intense and rapid genocide. The mortality rate for the Nazi killings of Jews in four camps and during shootings might indicate a less intense but longer genocide. The survival curve shows two rapid and intense periods of killings with a long period of stable survival probabilities in-between. Survival curves for Jewish men and women from the Netherlands indicate a smoother but also a longer killing process with a long tail of slightly decreasing survival rates. The higher survival probabilities for men till March 1944 and the drop in these probabilities thereafter might suggest men were more often selected for work when arriving at Nazi camps but these selections might not have been protective since men's survival rate equals that for women in the end (10).

The importance of epidemiological methods in natural disaster to identify determinants of mortality and in medical assessment of refugees fleeing mass killings was recognized in the 1970s and 1980s (13). Thereafter, epidemiologic methods were widely applied during humanitarian responses to the subsequent wave of African famines and postcolonial civil wars in the 1980s enabling health analysts to describe how mortality and morbidity differed across population groups and over time, providing crucial insights for improving response and preparedness (13). While in some other recent epidemiological contributions the emphasis is on data collection (14–16), this Opinion article focused on the use of a few measures to analyse the available data and thereby adding to the Haddon matrix (17) adapted for an epidemiological study of genocide by Adler et al. (18). Applying epidemiological measures, even with crude mortality data, might result in better or earlier identification or understanding of genocide-related death during potential future and current or imminent genocides such as those against Yazidis in Iraq (19), Rohingya Muslims in Myanmar (20), and Uyghurs and other Turkic Muslims in Northwest China (21), or the extreme sectarian violence between the pro-Muslim coalition Seleka and the pro-Christian movement Anti-Balaka in Central African Republic (22).

## Discussion

Tam et al. plead in their editorial “Epidemiology in conflict—A call to arms” for more structured research and reasoned discussion on wars' impact on the public health and possible interventions in future conflicts (23). Within the field of epidemiology, population and public health studies growing attention is given to deaths and sufferings related to homicide (24–26), domestic violence (27, 28), suicide (29, 30), and armed conflict (31–34); only a few epidemiological studies focused on genocide (10, 35, 36). This limited attention might be due to poorer data availability and quality. However, more data have become available over the past decades on, for example, the Holocaust.

Social science and historical studies focus mainly on causes of genocide, answering questions on “what,” “why,” and “how” it happened. Epidemiology, population, and public health studies should articulate the consequences of genocide, answering question on “who” became, “when,” and “where” victim. Applying epidemiological methods could result in better understanding of genocide-related deaths in the past while it also provides better opportunities for comparison of these deaths. This greater knowledge about historical genocide-related deaths might then contribute to the writing of national and local histories, and remembrance and commemoration by providing better context and patterns of victimization and killings. At the same time, it could contribute to reducing mortality in future genocide by providing data and quantitative information on death frequencies improving predictive and interventive models.

Quantifying Holocaust and other genocides using epidemiological measures not only contributes to our historical understanding of genocides, but also provides groundwork for the investigation of exposure to genocide on health and the intergenerational transmission of genocide-related trauma (37–41). Data such as the reported in this study can improve the precision of exposure definitions that use spatiotemporal information on the extent and severity of the exposure, which is especially valuable when individual-level data about the exposure status are not available.

Expanding our understanding of genocide-related deaths in the past, trying to prevent or reduce genocide-related deaths in the future, and improving health conditions of survivors and their offspring require input from different fields including epidemiology, population and public health studies.

## Author Contributions

The author confirms being the sole contributor of this work and has approved it for publication.

## Conflict of Interest

The author declares that the research was conducted in the absence of any commercial or financial relationships that could be construed as a potential conflict of interest.

## Publisher's Note

All claims expressed in this article are solely those of the authors and do not necessarily represent those of their affiliated organizations, or those of the publisher, the editors and the reviewers. Any product that may be evaluated in this article, or claim that may be made by its manufacturer, is not guaranteed or endorsed by the publisher.
